# Pre-hospital blood transfusion in non-traumatic major haemorrhage: a retrospective observational study

**DOI:** 10.1186/s13049-026-01556-x

**Published:** 2026-02-16

**Authors:** Shanshan Ru

**Affiliations:** Department of Pharmacy, Shaoxing Central Hospital, Shaoxing, China

Dear Editors

We read with great interest the article published in the *Scandinavian Journal of Trauma, Resuscitation and Emergency Medicine* entitled “Pre-hospital blood transfusion in non-traumatic major haemorrhage: a retrospective observational study” [1]. The authors address an important and underexplored area in pre-hospital emergency medicine, where current practice is frequently extrapolated from trauma literature. By providing a descriptive overview of real-world transfusion practice in non-traumatic haemorrhage, this study offers a valuable snapshot of current pre-hospital decision-making in a rare but high-risk clinical context. Nevertheless, several methodological considerations warrant caution in the interpretation of the study’s conclusions.

The primary evidence supporting clinical benefit is the reported reduction in shock index (SI) following transfusion. However, in a single-arm pre–post design, this finding is highly susceptible to regression to the mean (RTM), a well-recognised statistical phenomenon when patients are selected on the basis of extreme physiological values [2]. Because inclusion was driven by marked physiological derangement, subsequent improvement may occur independent of the intervention due to natural variability, compensatory responses, or spontaneous haemorrhage control—precisely the conditions under which RTM is most pronounced. In the absence of a contemporaneous control group, it is therefore not possible to disentangle any transfusion-specific effect from RTM or other non-specific influences, and the observed magnitude of improvement may overestimate any true treatment effect.

In addition, the use of SI as a surrogate endpoint warrants caution, particularly given the heterogeneity of bleeding aetiologies within the cohort. Improvements in surrogate physiological markers do not necessarily translate into patient-centred benefit, especially when survival remains poor, as has long been recognised in clinical trial methodology [3]. This concern is especially relevant in gastrointestinal haemorrhage, where validated risk stratification tools exist and SI has limited performance in predicting clinically meaningful outcomes [4]. In this study, despite reductions in SI, mortality among patients presenting in cardiac arrest remained extremely high, highlighting a potential dissociation between monitored physiological change and meaningful clinical outcomes.

The aggregation of pathophysiologically distinct conditions into a single analytical group further complicates interpretation. Non-traumatic haemorrhage encompasses entities with divergent haemodynamic goals, such as gastrointestinal bleeding, ruptured abdominal aortic aneurysm, and postpartum haemorrhage. Pooling these conditions risks obscuring important safety signals, as physiological targets appropriate for one aetiology may be harmful in another. Given the very small numbers within several subgroups, the study is underpowered to support even exploratory conclusions regarding safety or efficacy in these populations.

Finally, the low number of included cases over an extended study period suggests substantial selection bias. Only patients who survived long enough to receive HEMS intervention were eligible for inclusion, introducing immortal time bias, a recognised limitation of observational studies evaluating time-dependent interventions [5]. This necessarily excludes patients who died prior to HEMS arrival or who were deemed too stable to require transfusion, further limiting generalisability.

In conclusion, we appreciate the authors’ efforts to address a critical gap in pre-hospital emergency care, as non-traumatic major haemorrhage remains a high-stakes clinical scenario with limited tailored guidance. We believe the methodological considerations outlined above may help to further refine future research designs and enhance the rigor of evidence in this important field. These discussions are vital to ensuring that pre-hospital transfusion practices are both safe and effective for diverse patient populations. We look forward to continued advancements in understanding optimal transfusion strategies for non-traumatic haemorrhage.

## Data Availability

No datasets were generated or analysed during the current study.

## Abbreviations

HEMS: Helicopter Emergency Medical Service
RTM: Regression to the mean
SI: Shock index
